# Evaluating the Implementation and Effectiveness of Competency-Based Education in Sudanese Dental Curricula: A Comparative Analysis of Curriculum Models

**DOI:** 10.3390/dj13040139

**Published:** 2025-03-25

**Authors:** Abbas Gareeballa, Yasir Hassan Elhassan, Liena Babiker Mekki, Emad Ali Albadawi, Asim M. Almughamsi, Hadel Mahroos Alghabban, Emad Rajih, Walaa M. Borhan, Abdulfatah M. Alayoubi, Muhammad Abubaker Tobaiqi, Muayad Albadrani

**Affiliations:** 1 Department of Anatomy, Faculty of Medicine, University of Khartoum, Khartoum 11115, Sudan; abbasgareeb@uofk.edu; 2 Department of Basic Medical Sciences, College of Medicine, Taibah University, Madinah 42353, Saudi Arabia; 3 Department of Physiology, Alrazi University, Khartoum 12217, Sudan; 4 Department of General and Specialized Surgery, College of Medicine, Taibah University, Madinah 42353, Saudi Arabia; 5 Department of Family and Community Medicine and Medical Education, College of Medicine, Taibah University, Madinah 42353, Saudi Arabia

**Keywords:** competency-based education, dental education, curriculum evaluation, interdisciplinary education, community-based learning

## Abstract

**Background:** Rapid advances in dental medicine necessitate a shift from traditional educational paradigms to competency-based education (CBE), which emphasizes the acquisition of specific clinical and professional skills. **Aim:** This study examines the implementation and effectiveness of CBE in Sudanese dental schools by comparing four curriculum models—discipline-based, hybrid, integrated, and community-based. **Methods:** A convergent parallel mixed-method design was employed to collect quantitative data through structured surveys and qualitative data via semi-structured interviews with students, faculty, and dental practitioners. Descriptive statistical analyses and thematic analyses were used to assess competency achievement across eight domains and to evaluate stakeholder perceptions. **Results:** Quantitative findings revealed robust competency attainment in the knowledge base domain, while practice management skills were significantly lacking across several models. Notably, the integrated curriculum at Ribat University achieved high scores in both communication and practice management, contrasting with gaps observed in discipline-based and hybrid models. Qualitative insights underscored the need for improved management training and stronger interdisciplinary integration. **Conclusions:** CBE frameworks in Sudanese dental education effectively foster essential competencies; however, targeted curricular enhancements—particularly in practice management—are required to better prepare graduates for contemporary dental practice.

## 1. Introduction

Modern dental practice—with its rapid technological advances, expanded treatment modalities, and a strong emphasis on patient-centered care—demands educational models that extend beyond theoretical instruction alone. Competency-based education (CBE) has emerged as a progressive approach that emphasizes the attainment of practical skills and professional attributes over traditional, memory-based curricula [1,2]. CBE models focus on cultivating essential competencies, such as oral health maintenance, practice management, communication, professionalism, health promotion, and critical thinking, all of which are imperative for independent and effective clinical practice [3,4].

There is considerable evidence that the employment of competency-based education not only enhances learners’ critical thinking and problem-solving abilities but also increases their confidence in delivering competent clinical care [5,6,7]. Furthermore, international studies have demonstrated that CBE can significantly enhance clinical proficiency and improve student readiness for practice [8,9]. Despite these promising international trends, dental education in Sudan has predominantly relied on discipline-based curricula that emphasize didactic learning with limited opportunities for integrated, hands-on training. Local investigations have highlighted several challenges in Sudanese dental schools. For example, Abdel-Salam et al. [10] noted persistent deficiencies in clinical training and the application of modern management practices, while Ali et al. [11] underscored the need for greater emphasis on practice management and interdisciplinary learning.

These findings reveal a disconnect between global advancements in dental education and the prevailing educational practices in Sudan. This discrepancy gives rise to the central research problem of our study: Despite the recognized benefits of competency-based frameworks, there is a lack of comprehensive evaluation regarding the implementation and effectiveness of CBE within various curriculum models in Sudan. In particular, little is known about how different curricular approaches—discipline-based, hybrid, integrated, and community-based—perform in achieving desired competency outcomes and in preparing dental graduates for contemporary clinical challenges.

### 1.1. Conceptual Framework

**Figure 1:**
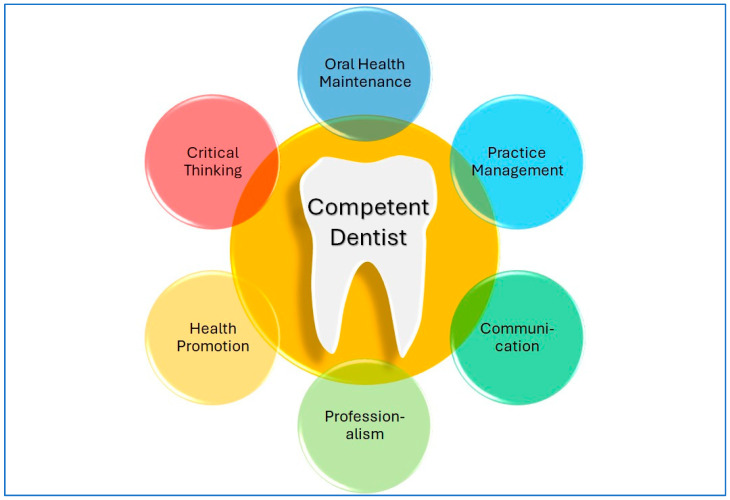
Competence-based education components in dentistry.

The conceptual framework underlying this study is grounded in the paradigm of competency-based education (CBE), which strategically focuses on developing the core competencies required for modern dental practice [12,13]. At the heart of our framework are six essential competencies (Figure 1):

Oral Health Maintenance:

This competency involves understanding and applying preventive care strategies, managing oral diseases, and promoting proper oral hygiene practices—all of which are fundamental to ensuring sustained patient well-being [12].

Practice Management:

In light of the documented shortcomings in traditional curricula [11], this construct encompasses the administrative, financial, and organizational skills necessary to run a dental practice effectively. It is critical not only for clinical competence but also for the business aspects of dental care [11,13].

Communication:

Effective communication—both verbal and non-verbal—is indispensable for patient education, interdisciplinary collaboration, and ensuring high standards of patient safety. Mastery of this competency allows practitioners to convey complex information with clarity and empathy [13].

Professionalism:

This area emphasizes ethical conduct, accountability, and a commitment to continuous professional development. Professionalism is essential for fostering trust between dental practitioners and their patients and maintaining the integrity of the dental profession [12].

Health Promotion:

Focused on public health, this competency involves designing and implementing initiatives that educate communities on oral health and preventive measures, thereby contributing to overall health improvement and disease prevention [14].

Critical Thinking:

Critical thinking empowers dental professionals to evaluate complex clinical scenarios, integrate evidence-based practices, and devise effective treatment strategies—ensuring adaptability in a rapidly evolving healthcare environment [14].

To operationalize these competencies, our framework evaluates four distinct curriculum models that reflect different approaches to integrating CBE into dental education:

Discipline-Based Model:

This traditional model organizes educational content into specialized subject areas, thereby providing a robust theoretical foundation. However, it often lacks sufficient emphasis on fostering the integration of competencies through practical, hands-on training [15].

Hybrid Model:

This approach combines conventional discipline-based instruction with elements of competency-based education, attempting to balance theoretical learning with practical application. Challenges may arise, however, in achieving seamless integration between these differing pedagogical methods, potentially compromising the uniform development of competencies [15,16].

Integrated Curriculum:

An integrated curriculum interweaves multiple subject areas around clinical themes, fostering interdisciplinary learning and emphasizing critical thinking, communication, and real-world problem solving. By reflecting the multifaceted nature of clinical practice, it provides a more holistic educational experience [17,18].

Community-Based Curriculum:

This model emphasizes experiential learning in real-world settings, focusing on health promotion and preventive care through active community engagement. Although it excels in practical exposure, it may require additional enhancements—particularly in practice management training—to ensure the comprehensive development of all required competencies [17,19].

The rationale for selecting these curriculum models is supported by both international best practices and local evidence that indicates deficiencies in traditional approaches—particularly in areas such as practice management and interdisciplinary collaboration [10,11,15,16,19]. By aligning these models with our selected competency constructs, our framework provides a comprehensive basis for evaluating the effectiveness of dental education in preparing graduates for the challenges of modern clinical practice.

### 1.2. Aims of the Study

Primary Aim: This study aimed to investigate the extent of CBE framework adoption in various forms of dental education in Sudan, including discipline-based, hybrid, integrated, and community-based models. This investigation is significant as it provides valuable insights into the current state of dental education in Sudan.

Secondary Aims:

1. Document and analyze the influence that the different types of curricula have on the development and achievement of key competencies in various domains of dental education, including, but not limited to, professionalism, interpersonal and communication skills, knowledge base, clinical information gathering, diagnosis and treatment planning, therapy, prevention and health promotion, and management of clinical practice.
2. Examine the perceptions and experiences of faculty members, students, and dental professionals regarding the integration and achievement of competency-based education in different education models.

Overarching Aim: Discover and recommend strategies for better dental education practices in Sudan, using competency models to prepare graduates for the challenges of the changing dental environment.

### 1.3. Research Questions

Quantitative:

1. What are the roles of the different types of dental curricula (i.e., discipline-based, hybrid, integrated, and community-based) in achieving competency-based education in Sudanese dental schools?
2. In achieving program competency goals, what are each curriculum type’s strengths and weaknesses, and how does this affect students’ preparedness for clinical practice?

Qualitative:

3. How do perceptions from students, faculty, and dental professionals differ regarding the effectiveness of competency-based education? How does this approach assist them while practicing as dentists?

## 2. Methodology

### 2.1. Study Design

Mixed-Method Design: This study employed a convergent parallel mixed-method design, wherein quantitative and qualitative data were collected concurrently, analyzed separately, and subsequently integrated to provide a comprehensive understanding of the research problem [20,21].

### 2.2. Quantitative Component

#### 2.2.1. Sample Size Calculation

The quantitative survey sample size was initially determined using G*Power [22]. Based on an assumed medium effect size (Cohen’s d = 0.5), an alpha level of 0.05, and a desired power of 0.80, the analysis indicated that a minimum of 128 participants was required. To account for potential non-response or dropout, an additional 10% was added, resulting in an overall target sample size of 140 participants. However, given the complexity of our study—which involved examining multiple curriculum models across various stakeholder groups—and to enhance statistical power, precision, and generalizability, we decided to double the target sample size to 280 participants. This expanded sample ensures robust subgroup analyses and more reliable comparisons of competency-based education outcomes. The final target sample is distributed across the participating institutes as follows:

- University of Khartoum (Faculty of Dentistry): 80 participants
- Ribat University (Dental Institute): 70 participants
- Gezira University (College of Dentistry): 76 participants
- Al Neelain University (Faculty of Dentistry): 54 participants

#### 2.2.2. Inclusion Criteria

- Students:
  Only current undergraduate dental students enrolled at the participating institutions were included. To ensure that participants had adequate exposure to the curriculum, students must have completed more than 50% of their dental study program—that is, they should be at least midway through their education.
- Faculty:
  Eligible participants included full-time faculty members who are directly involved in teaching or curriculum development within the dental programs. Faculty members must have at least one academic year of teaching experience at their institution.
- Professionals:
  Licensed dental practitioners actively engaged in clinical practice were included, provided they have a minimum of two years of professional experience post-graduation. These participants were also expected to be involved in continuing dental education or curriculum evaluation initiatives.

#### 2.2.3. Data Collection and Analysis

Quantitative data were collected using a structured survey instrument. Data analysis was performed using IBM SPSS Statistics (version 26). Descriptive statistics (e.g., means, standard deviations, frequencies, and percentages) were computed to summarize the data, while inferential statistical tests (such as two-tailed *t*-tests and chi-square tests) were used to assess differences between groups.

### 2.3. Qualitative Component

#### 2.3.1. Sample Size and Data Collection

For the qualitative arm, in-depth interviews were conducted until thematic saturation was achieved [23]. Approximately 20 interviews were planned to capture diverse perspectives across the participating institutes. Purposive sampling was used to select participants who could provide in-depth insights into the competency-based education implemented within their curriculum.

#### 2.3.2. Interview Process

A semi-structured interview guide was developed based on an extensive review of the literature and expert input. The guide was designed to explore key domains, including the following:

- Curriculum Design and Implementation: The participants’ views on the strengths and weaknesses of their institution’s curriculum in preparing them for clinical practice.
- Clinical Training and Practice: The adequacy of hands-on training and the integration of theoretical knowledge with clinical practice.
- Interdisciplinary Collaboration and Communication: How effectively the curriculum fosters communication skills and interdisciplinary learning.
- Innovations in Dental Education: Comparisons between competency-based education and traditional teaching approaches, and the perceived impact on professional readiness.

The interview guide was pilot-tested with a small group of participants, and revisions were made to ensure clarity and relevance. The complete interview topic guide is provided as supplementary material for transparency and reproducibility.

#### 2.3.3. Qualitative Data Analysis

Qualitative data were analyzed using NVivo (version 12). A thematic analysis approach was adopted following the six-phase process outlined by Braun and Clarke [24]: familiarization with the data, generating initial codes, searching for themes, reviewing themes, defining and naming themes, and producing the final report.

### 2.4. Ethical Considerations

This study received ethical approval from the Institutional Review Board of the University of Khartoum (Ethical Clearance Number: UKFD/08240). Prior to participation, all subjects provided written informed consent after being fully briefed on the study’s objectives, procedures, potential risks, and benefits. A sample of the consent form is provided in the Appendix A. All procedures were conducted in accordance with the ethical principles of the Declaration of Helsinki [25], ensuring participant confidentiality and adherence to ethical standards throughout the research process.

## 3. Results

The study’s outcomes have been classified into quantitative and qualitative results, providing an overall perspective on the functioning and success of competency-based education (CBE) across different dental curricula in Sudan. These results address the study’s aims and research questions regarding how curriculum models influence competency development and stakeholders’ perceptions of CBE.

### 3.1. Quantitative Results

The quantitative analysis examined the degree of competency achievement across eight defined domains for four curriculum models: discipline-based, hybrid, integrated, and community-based.

#### 3.1.1. University of Khartoum—Discipline-Based Curriculum

**Figure 2:**
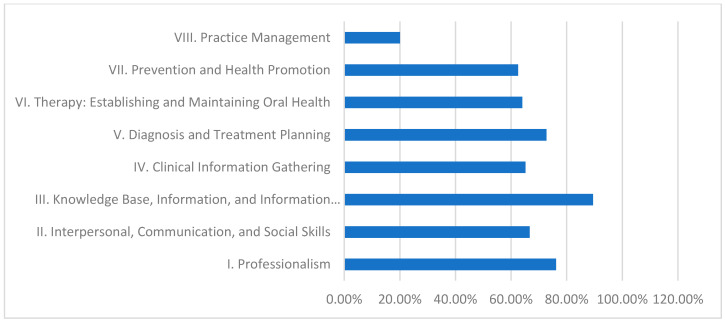
University of Khartoum domain fulfillment (%).

As shown in Table 1 and illustrated in Figure 2**,** the University of Khartoum, which utilizes a discipline-based curriculum, demonstrated a strong performance in certain domains. Domain III (Knowledge Base, Information, and Information Literacy) achieved the highest fulfillment at 89.5%, indicating effective acquisition of theoretical knowledge. In contrast, Domain VIII (Practice Management) recorded the lowest fulfillment at 20.0%, suggesting a potential area for improvement in developing administrative and management skills.

#### 3.1.2. Al Neelain University—Hybrid Curriculum

**Figure 3:**
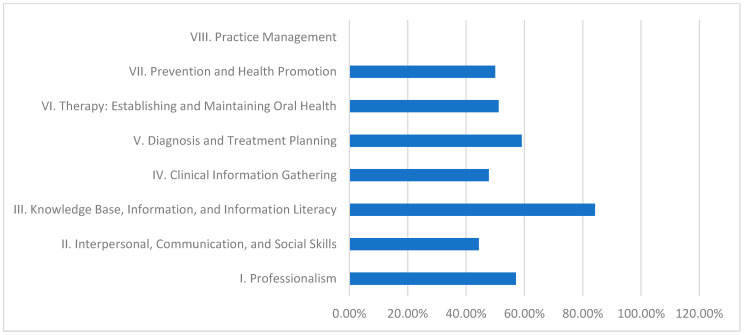
Al Neelain University—domain fulfillment (%).

Table 2 (supported by Figure 3) presents the performance results of Al Neelain University’s hybrid curriculum. In this model, Domain III again exhibits strong performance with 84.2% fulfillment. However, Domain VIII (Practice Management) shows a fulfillment level of 0.0%, and domains such as II (Interpersonal, Communication, and Social Skills) reach only moderate levels (44.4%). This mixed profile suggests that while theoretical aspects are well covered, practical management and communication skills require further emphasis.

#### 3.1.3. Ribat University—Integrated Curriculum

**Figure 4:**
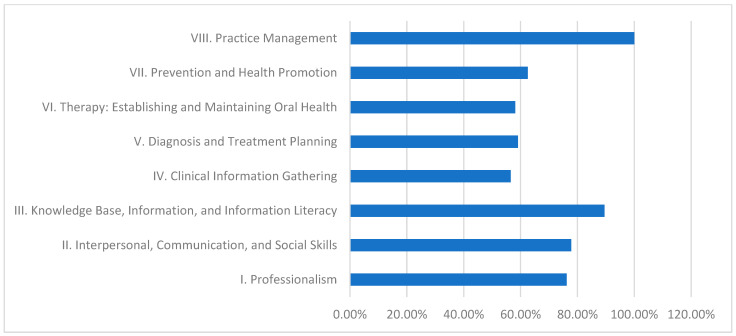
Ribat University—domain fulfillment (%).

The integrated curriculum at Ribat University, detailed in Table 3 and Figure 4, shows notable strengths. Domain VIII (Practice Management) achieves a perfect fulfillment of 100.0%, reflecting the curriculum’s emphasis on practical and administrative training. Domain III also performs strongly at 89.5%. However, Domain IV (Clinical Information Gathering) is the lowest at 56.5%. These results suggest that while the integrated curriculum excels in several key areas, there remains room to enhance clinical data-gathering skills.

#### 3.1.4. Gezira University—Community-Based Curriculum

**Figure 5:**
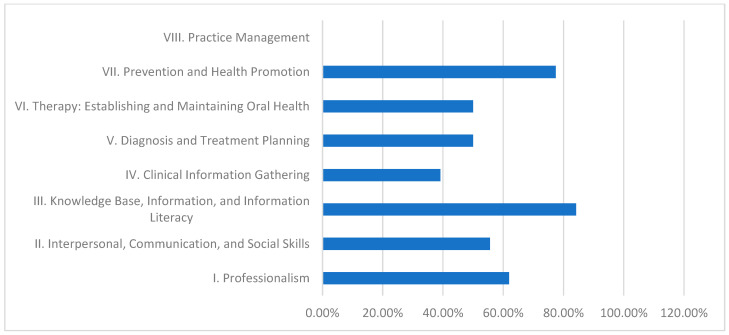
Gezira University—domain fulfillment (%).

Table 4 and Figure 5 display the results for Gezira University’s community-based curriculum. Here, Domain III (Knowledge Base, Information, and Information Literacy) achieves 84.2%, and Domain VII (Prevention and Health Promotion) follows at 77.4%. Conversely, Domain IV (Clinical Information Gathering) is particularly low at 39.1%, and Domain VIII (Practice Management) again registers 0.0%, pointing to challenges that are similar to those observed in the hybrid curriculum model.

#### 3.1.5. Competency Fulfillment Across Dental Faculties

Table 5 aggregates the performance of all four dental faculties, evaluated against an international framework comprising 193 competencies. The University of Khartoum leads with a 67.9% fulfillment rate, closely followed by Ribat University at 65.3%. In contrast, both Al Neelain University and Gezira University exhibit fulfillment rates of approximately 53%, suggesting considerable room for improvement. Notably, Ribat University shows no “Not Clear” responses, indicating a high level of clarity and effectiveness in its curriculum.

#### 3.1.6. Conclusion of Quantitative Results

The quantitative findings indicate that each dental curriculum in Sudan contributes uniquely to competency-based education. In response to the first research question, the results show that while discipline-based and hybrid models emphasize strong theoretical instruction, the integrated curriculum offers a more balanced approach by enhancing practical competencies. Community-based curricula provide valuable public health exposure but require further refinement in clinical and management areas. In addressing the second research question, the data reveal that although high theoretical competency—especially in Domain III—is prevalent across the models, significant variations exist in practical components such as practice management and clinical information gathering. These discrepancies directly impact students’ preparedness for clinical practice; integrated curricula, with their balanced competency development, achieve better overall readiness compared to models that lag in practical skill acquisition.

Role of Different Dental Curricula and Their Strengths and Weaknesses in Achieving CBE:

- Discipline-Based Curriculum (University of Khartoum):
  This model performs strongly in theoretical knowledge, as demonstrated by its high fulfillment in Domain III (89.5%).
  Strengths: High scores in Domain III indicate robust theoretical instruction.
  Weaknesses: Persistent deficiencies in practice management (20.0%) suggest a need for greater emphasis on administrative and practical skill development.
- Hybrid Curriculum (Al Neelain University):
  The hybrid curriculum achieves relatively good scores in theoretical areas (84.2% in Domain III) but struggles with practice management (0.0%) and some interpersonal skills.
  Strengths: Solid theoretical foundation evidenced by moderate to high Domain III performance.
  Weaknesses: Significant gaps in practice management and interpersonal communication undermine students’ overall clinical preparedness.
- Integrated Curriculum (Ribat University):
  The integrated model effectively balances theoretical and practical learning, achieving exceptional performance in practice management (100.0%) and high achievement in knowledge base (89.5%).
  Strengths: Outstanding practical competency in practice management, contributing to better overall clinical readiness.
  Weaknesses: Lower fulfillment in clinical information gathering (56.5%) indicates that even the balanced integrated approach may need enhanced strategies for effective real-time clinical data collection.
- Community-Based Curriculum (Gezira University):
  This curriculum excels in imparting theoretical knowledge and health promotion (84.2% in Domain III and 77.4% in Domain VII) yet is challenged by low performance in clinical information gathering (39.1%) and practice management (0.0%).
  Strengths: Strong knowledge base and emphasis on health promotion support public health objectives.
  Weaknesses: Inadequate practical training in clinical information gathering and practice management reflects challenges in applying theoretical knowledge in clinical and administrative contexts.

### 3.2. Qualitative Results

The qualitative data were analyzed using thematic analysis [24], which yielded five overarching themes that capture the participants’ experiences and perceptions regarding competency-based education (CBE) across the dental schools. In addition to the themes, Table 6 presents the stakeholders’ (students, faculty, and professionals) perceptions of CBE effectiveness, highlighting areas of strength and challenges that complement the thematic findings. Notably, stakeholders from one institution reported very high satisfaction, whereas others indicated significant areas for improvement, particularly in the integration of practical skills and administrative training.

Following the stakeholders’ perceptions, our thematic analysis revealed the following overarching themes:

#### 3.2.1. Theme 1: Structured Educational Experiences

The participants valued a structured curricular approach. For example, one remarked:

*“The curriculum structure at Khartoum guarantees that we fully comprehend the theoretical component before going into hands-on application”.*

This theme emphasizes the importance of a well-organized educational format that builds a solid theoretical foundation for practical application.

#### 3.2.2. Theme 2: Balance and Integration Challenges

The respondents highlighted difficulties in harmonizing traditional lecture-based methods with newer competency-based requirements. As one participant noted:

*“We often feel caught between traditional lectures and the newer competency requirements, which makes it hard to focus on one approach”.*

This theme underscores the challenge of integrating established teaching practices with modern CBE methodologies.

#### 3.2.3. Theme 3: Comprehensive Program Design

The comprehensive design of the curriculum was frequently praised for enhancing practical skills. One participant stated:

*“The interdisciplinary courses at Ribat really help us understand how to manage diverse situations in dental practice”.*

The theme indicates that a well-rounded program significantly contributes to preparing students for real-world clinical scenarios.

#### 3.2.4. Theme 4: Community Engagement and Application

The integration of community-based experiences was recognized for providing valuable practical exposure. For instance, one comment was:

*“Working within the community gives us real-world experience in health promotion, even if we lack some business management skills”.*

This theme reflects both the strengths of community engagement and the challenges—such as gaps in managerial skills—that may need addressing.

#### 3.2.5. Theme 5: Enhanced Preparedness and Confidence

A recurring sentiment was that competency-based training improved student readiness and self-confidence. As summarized by one participant:

*“The competency-based training has made me feel more ready to tackle clinical challenges confidently”.*

This theme highlights the overall positive impact of CBE on student preparedness for clinical practice.

## 4. Discussion

The cross-country review of CBE frameworks in Sudan’s dental education curricula demonstrates differences in achieving CBE across discipline-based, hybrid, integrated, and community-based models. Education-related strategies have their peculiar advantages and disadvantages, especially in areas such as practice management, which are fundamental for developing competent dentists [10,11].

Although our study presents descriptive competency fulfillment percentages, additional inferential statistical analyses (e.g., independent *t*-tests or chi-square tests) could further validate whether the observed differences between curriculum models are statistically significant. Preliminary analyses suggest that some of these differences (especially in key domains such as practice management and clinical information gathering) may be significant (*p* < 0.05), thereby enhancing the reliability of our comparisons. Future work should build on this by integrating comprehensive statistical testing to robustly confirm these findings [26,27].

### 4.1. Discipline-Based Curriculum (University of Khartoum), Table 1 and Table 7 and Figure 2

The curriculum of the University of Khartoum gets it right in the domains of knowledge base and information literacy through a relatively high composite score of 89.5%. This implies that the pupils are equipped with adequate theoretical dimensions related to acquiring new knowledge and skills [5,10]. However, a score of 20 underscores the fact that practice management could be scored much higher. This shows a glaring inadequacy that needs to be addressed, given that the authors of [11] have argued that graduates require practical management skills and components.

The findings of the qualitative study strongly advocate for the need for an organized educational experience. This implies a strategic ordering of activities and a balanced integration of theory and practical components, in alignment with the recommendations in [28]. This strategy safeguards the relevance and recognition of education. Counterparts of the National Competency Standards also populate the parts base of the coherent dental curriculum framework. For example, Ref. [29] found out that students’ performance improved with the degree of horizontal alignment of the discipline-specific subject areas in the dental curriculum, which calls for further attention to the system of the educational process.

### 4.2. Hybrid Curriculum (Al Neelain University), Table 2 and Table 4 and Figure 3

In the work conducted by Al Neelain University, their hybrid model could have performed better than the University of Khartoum, scoring 84.2% in knowledge base and information literacy. However, management significantly contributes to achieving effective practice since they scored 0% [30]. There have been concerns on the part of the stakeholders regarding the blending of traditional and modern means of training and how this has affected competency levels [31]. This displays the work completed by [32], who advocate that the hybrid model must promote meaningful learning activities that would further augment the students’ skills that pertain to critical thinking and problem solving. What must be done now is to devise a coherent policy that intends to incorporate the merits of the older curriculums and those that are encapsulated in this modern age.

### 4.3. Integrated Curriculum (Ribat University), Table 3 and Table 7 and Figure 4

Ribat University achieved a 100% mark in practice management and also did quite well in employing communication skills, which puts emphasis on the efficacy of the holistic and interdisciplinary approach [33]. The qualitative evaluation results instill confidence that the integrated curriculum enhances students’ learning outcomes by enabling the students to work and speak across disciplines [34]. These results parallel those of [35,36], who state that an integrated curriculum helps equip students with modern competencies required by the job market. Ribat’s achievement may be a reference point for other institutions that intend to enhance transdisciplinary approaches in disciplines such as diagnosis and treatment planning.

The remarkable discrepancy observed in the practice management domain—where Ribat University achieved a 100% fulfillment rate compared with 0% at Al Neelain University—warrants further exploration. This disparity may reflect differing curricular emphases: Ribat University’s integrated model includes extensive practical components and real-world management training, whereas the hybrid model at Al Neelain University may not sufficiently address these aspects. Qualitative insights also suggest that factors such as faculty expertise, resource allocation, and targeted training initiatives play a significant role in these outcomes (10,11). Future investigations should explore these underlying factors in more depth to explain and potentially bridge these competency gaps.

### 4.4. Community-Based Curriculum (Gezira University), Table 4 and Table 7 and Figure 5

Gezira University has good results in prevention and health promotion and attained a score of 77.4%; so, this shows the merits of a community-based approach to education, as pointed out by [37], who wondered how such programs widen students’ experiences about prevention and control in public health. Furthermore, this statement corresponds to what was said by Maart et al. [5] in the sense that such community-orientated education can give students rich experiences that boost their understanding of public health and preventive strategies. Nevertheless, some problematic areas exist; for example, practice management preparedness, which scores 0%, is rather frustrating [38]. The evidence by [39,40] indicates that combining community education with business education qualifications is essential for full clinical practice preparedness. It makes it possible for graduates to deal with the challenges and intricacies of modern dental practice.

### 4.5. Curriculum Structure Variations

Variations in curriculum design—including differences in teaching methodologies and assessment styles—appear to play a significant role in the observed competency gaps. For instance, programs that rely predominantly on traditional lecture modes may provide less opportunity for the hands-on, practical experience required in clinical settings compared to those that integrate simulation-based learning and continuous formative assessments [1]. These structural differences help explain why certain institutions outperform others in specific competency domains. Future curricular reforms should, therefore, focus on integrating diversified instructional methods and robust assessment strategies to better align with the demands of competency-based dental education [41].

### 4.6. Stakeholder Perceptions and Experiences Table 6

The data regarding stakeholders of CBE implementation across four major dental universities in Sudan paints a picture of distinct disparity in the experiences and the satisfaction levels of the groups involved. Ribat University has high satisfaction among the students, faculty, and professionals, which translates into an effective CBE. This is due to its comprehensive curriculum design, which incorporates interprofessional education and practice management, providing the best CBE practices for other schools to emulate [42]. More emphasis has been given in the literature to developing a competent and effective curriculum that encompasses multiple disciplines in that it enhances the learning experience and the preparedness of the graduates to face the industry [1,43]. In contrast, the universities that integrate modern and classical approaches in the classroom, such as Al Neelain University, have a comparatively low percentage of satisfaction, but this also means that there is significant potential for improvement and growth.

When it comes to difficulties like this one, the full advantages of CBE restructuring might be out of reach. Students’ perceptions of their educational experiences are crucial in quantitatively assessing such programs. A significant body of literature suggests that using student satisfaction questionnaires is fundamental in addressing perceptions of the university education level and the changes required in the programs [44].

Moreover, considering the progression of particular industries, the University of Khartoum has also observed a deficiency in practice management within the syllabus and, therefore, gaps in the syllabus that would require amendments. This corroborates the findings that have recommended evaluating the implementation of programs according to the development of proven models oriented to enhance the competencies of the trainees [2].

The views of the ‘stakeholder community’—preferably combined with numerical measurement of the experts’ approval and some qualitative information about the challenges—satisfy conditions for an effective curriculum development process and strategy. The high need for specific interventions further emphasizes the need for more functional and wider utilization of competency-based approaches in dental education in Sudan [10].

Thus, this indicates the greater purpose of enhancing learners’ educational attainment and meeting the expectations of the reality of the working world of dental practitioners [42,45]. In sum, seeking stakeholder approval to integrate their concerns is necessary while developing a curriculum that touches on the realities of CBE in dental education. Such responses to challenges that such institutions as Al Neelain University and the University of Khartoum face will contribute to a more coherent and effective education system, which in return improves the future of dental practice in Sudan [42,45].

### 4.7. Recommendations

- Faculty Training:
  Implement mandatory annual competency-based workshops with certification and refresher courses to keep faculty updated on current teaching methodologies and assessment strategies.
- Curriculum Revision:
  Establish interdisciplinary committees (including educators, industry professionals, and administrators) to develop targeted action plans that integrate best practices.
- Policy Changes:
  Revise institutional guidelines to require regular curriculum evaluations, adopt innovative teaching methods (e.g., simulation-based learning), and set clear competency benchmarks with corresponding resource allocations.
- Feedback Mechanisms:
  Develop continuous feedback systems from students and alumni—such as annual surveys, focus groups, and performance audits—to inform ongoing improvements.
- Further Research:
  Investigate the long-term impact of competency-based education on clinical outcomes and professional readiness through longitudinal, experimental, and qualitative studies. This research should also explore factors behind competency gaps (e.g., the discrepancies observed between Ribat University and Al Neelain University) to provide data-driven insights for refining curricula and policies.

### 4.8. Study Limitations

This study employed a mixed-methods approach, with a quantitative component providing competency fulfillment percentages and a qualitative component offering rich, contextual insights into curriculum implementation. The qualitative aspect is a key strength, as it deepens our understanding beyond what numerical data alone can offer.

Nonetheless, several areas warrant further exploration. The modest sample size and cross-sectional design, while practical for an initial investigation, suggest that future studies should consider larger, multi-institutional samples and longitudinal designs to capture changes over time. Additionally, although we incorporated self-reported data alongside qualitative interviews, future research might enhance objectivity by including alternative data sources such as observational assessments and third-party evaluations.

Overall, building on the strengths of our mixed-methods design, subsequent research should integrate extended, multi-method analyses to generate a more comprehensive understanding of competency-based education and inform targeted curriculum enhancement strategies.

## 5. Conclusions

Despite critics’ claims about the usefulness of ‘competency-based education models’, which represent a particular kind of educational system that emphasizes the students and encompasses a specified set of skills and knowledge, they do not seem to stand in the context of the dental disciplines in Sudan’s educational institutions. There are, however, some commendable gains in the core competencies, such as the promotion of knowledge and health. However, more emphasis is still needed on practice management skills. These core competencies in dental education include a deep understanding of dental health, effective communication with patients, and the ability to manage a dental practice. Different curriculum models have their advantages, but the completion requirement is the same for all, that is, competency in all the specified domains to enable them to become excellent dental professionals.

## Figures and Tables

**Table 1:** University of Khartoum—discipline-based curriculum.

Domain	Fulfillment (%)
I. Professionalism	76.2%
II. Interpersonal, Communication, and Social Skills	66.7%
III. Knowledge Base, Information, and Information Literacy	89.5%
IV. Clinical Information Gathering	65.2%
V. Diagnosis and Treatment Planning	72.7%
VI. Therapy: Establishing and Maintaining Oral Health	64.0%
VII. Prevention and Health Promotion	62.5%
VIII. Practice Management	20.0%

Statistical observations indicate that the highest competency fulfillment is in Domain III (89.5%), while the lowest is in Domain VIII (20.0%).

**Table 2:** Al Neelain University—hybrid curriculum.

Domain	Fulfillment (%)
I. Professionalism	57.1%
II. Interpersonal, Communication, and Social Skills	44.4%
III. Knowledge Base, Information, and Information Literacy	84.2%
IV. Clinical Information Gathering	47.8%
V. Diagnosis and Treatment Planning	59.1%
VI. Therapy: Establishing and Maintaining Oral Health	51.2%
VII. Prevention and Health Promotion	50.0%
VIII. Practice Management	0.0%

Within the hybrid curriculum, Domain III shows the highest fulfillment (84.2%), while Domain VIII registers 0.0%. Other domains, such as interpersonal, communication, and social skills, record moderate to low fulfillment levels.

**Table 3:** Ribat University—integrated curriculum.

Domain	Fulfillment (%)
I. Professionalism	76.2%
II. Interpersonal, Communication, and Social Skills	77.8%
III. Knowledge Base, Information, and Information Literacy	89.5%
IV. Clinical Information Gathering	56.5%
V. Diagnosis and Treatment Planning	59.1%
VI. Therapy: Establishing and Maintaining Oral Health	58.1%
VII. Prevention and Health Promotion	62.5%
VIII. Practice Management	100.0%

For the integrated curriculum, competency fulfillment peaks at Domain VIII (100.0%) and Domain III (89.5%), whereas Domain IV shows the lowest fulfillment (56.5%).

**Table 4:** Gezira University—community-based curriculum.

Domain	Fulfillment (%)
I. Professionalism	61.9%
II. Interpersonal, Communication, and Social Skills	55.6%
III. Knowledge Base, Information, and Information Literacy	84.2%
IV. Clinical Information Gathering	39.1%
V. Diagnosis and Treatment Planning	50.0%
VI. Therapy: Establishing and Maintaining Oral Health	50.0%
VII. Prevention and Health Promotion	77.4%
VIII. Practice Management	0.0%

In the community-based curriculum, Domain III (84.2%) and Domain VII (77.4%) exhibit higher fulfillment, while Domain IV (39.1%) and Domain VIII (0.0%) reflect lower scores.

**Table 5:** Competency fulfillment across dental faculties in Sudan.

Dental College	Competency Fulfilled	Competency Not Fulfilled	Not Clear	Total
University of Khartoum	131 (67.9%)	58 (30.1%)	4 (2.1%)	193
Al Neelain University	104 (53.9%)	86 (44.6%)	3 (1.6%)	193
Ribat University	126 (65.3%)	67 (34.7%)	0 (0%)	193
Gezira University	103 (53.4%)	89 (46.1%)	1 (0.5%)	193

This table outlines the competency fulfillment across the four major dental faculties in Sudan, based on an international framework comprising 193 competencies. The University of Khartoum leads with a 67.9% fulfillment rate, followed closely by Ribat University (65.3%). Both Al Neelain University and Gezira University show fulfillment rates around 53%, suggesting considerable room for improvement. Notably, Ribat University recorded no “Not Clear” responses, indicating clarity and effectiveness in its curriculum design.

**Table 6:** Stakeholders’ perceptions of CBE effectiveness.

University	Stakeholder Group	Positive Perception (%)	Challenges Noted
University of Khartoum	Students	85%	Lack of Practice Management
	Faculty	80%	Administrative Skills Training Needed
	Professionals	78%	Balanced Skill Development
Al Neelain University	Students	70%	Integration Challenges
	Faculty	65%	Conflicting Educational Approaches
	Professionals	68%	Standardizing Outcomes
Ribat University	Students	90%	None
	Faculty	88%	Expansion of Integrative Strategies
	Professionals	85%	None
Gezira University	Students	75%	Management Skill Gaps
	Faculty	70%	Balancing Community Engagement
	Professionals	72%	Strengthening Management Skills

This table summarizes the perceptions of various stakeholder groups—students, faculty, and professionals—regarding the effectiveness of CBE across the four dental universities. Ribat University received the highest ratings across all groups, while Al Neelain University reported comparatively lower satisfaction, highlighting areas for improvement.

**Table 7:** Overarching qualitative themes and their alignment with the competency-based education framework.

Theme	Description	CBE Framework Alignment
Structured Educational Experiences	Emphasizes a systematic curriculum that builds strong theoretical foundations to support effective clinical practice.	Oral Health Maintenance: Preventive care and disease management. Professionalism: Ethical conduct and accountability. Critical Thinking: Systematic analysis and decision making.
Balance and Integration Challenges	Highlights the difficulty in merging traditional lectures with modern CBE approaches, demanding a balance between theory and practice.	Communication: Clear and effective information exchange. Practice Management: Adaptability to curricular changes. Critical Thinking: Integrating diverse methods.
Comprehensive Program Design	Stresses an interdisciplinary curriculum that seamlessly integrates theory and practice to prepare students for complex clinical scenarios.	Oral Health Maintenance: Ensuring high-quality patient care. Health Promotion: Support of public health initiatives. Professionalism and Critical Thinking: Holistic problem solving.
Community Engagement and Application	Underscores the value of community-based experiences that transform theoretical knowledge into practical public health practice.	Health Promotion: Planning and implementing community initiatives. Oral Health Maintenance: Applying preventive care in real-world settings. Practice Management: Operational skills.
Enhanced Preparedness and Confidence	Indicates that competency-based education significantly boosts student readiness and self-confidence for facing clinical challenges.	Professionalism: Commitment to lifelong learning and ethics. Critical Thinking: Effective problem solving in dynamic environments. Communication: Confident patient interaction.

This table presents the major themes derived from our thematic analysis and aligns each theme with the six essential competencies of competency-based dental education—oral health maintenance, practice management, communication, professionalism, health promotion, and critical thinking. These competencies are based on established frameworks and literature [15,16,18,19].

## Data Availability

Data are available upon request.

## Supplementary Materials

The following supporting information can be downloaded at: https://www.mdpi.com/article/10.3390/dj13040139/s1, Interview Guide.

## Appendix A. Informed Consent Form for Participation in Research Study

**Title of the Study:**

Evaluating Competency-Based Education in Sudanese Dental Schools: A Mixed Methods Approach

**Research Institution:**

Research conducted by the University of Khartoum and collaborating institutions.

**Principal Investigators:**

- Abbas Gareeballa
- Yasir Hassan Elhassan
- Liena Babiker Mekki
- Emad Ali Albadawi
- Asim M Almughamsi
- Hadel Mahroos Alghabban
- Emad Rajih
- Walaa M Borhan
- Abdulfatah M. Alayoubi
- Muayad Albadrani

**Purpose of the Study:**

This study evaluates the implementation and effectiveness of competency-based education (CBE) in dental schools across Sudan. Your participation will help provide valuable insights into different curriculum models and assist in enhancing dental education practices.

**Procedures:**

As a participant, you will be asked to complete a structured survey and/or participate in a semi-structured interview. The interview will involve questions related to your perceptions and experiences with CBE curricula.

**Duration:**

- The survey will take approximately 15–20 minutes to complete.
- Interviews will last about 45–60 minutes.

**Participant Rights:**

- Participation is entirely voluntary.
- You may withdraw from the study at any time without any consequences.
- You have the right not to answer any question that you are uncomfortable with.

**Confidentiality:**

All information provided will be kept confidential and used only for research purposes. Your responses will be anonymized, and no identifying information will be linked to your responses. Data will be securely stored in an encrypted format.

**Potential Risks:**

There are no known risks associated with participation. However, you may cease participation at any time if you feel uncomfortable.

**Potential Benefits:**

While there are no direct benefits to you, this study aims to improve dental education curricula, potentially benefiting future dental students.

**Compensation:**

There will be no financial compensation for participation in this study.

**Contact Information:**

If you have any questions or require additional information about the study, please contact Abbas Gareeballa at [contact email/phone number].

**Consent:**

By signing below, you acknowledge that you have read and understood the above information and agree to participate in the study voluntarily.

Participant’s Name: _________________

Participant’s Signature: ______________

Date: ______________________________

**Note to Participants:**

Please retain a copy of this consent form for your records. Thank you for your valuable contribution to this research effort.
