# Supplementary material for: Evaluating the Implementation and Effectiveness of Competency-Based Education in Sudanese Dental Curricula: A Comparative Analysis of Curriculum Models
Source: Dent J (Basel). 2025 Mar 25;13(4):139. doi: 10.3390/dj13040139 (PMC12025711; doi:10.3390/dj13040139)
Supplement: Supplementary file 1 [file dentistry-13-00139-s001.zip › dentistry-3477271-supplementary materials_Interview Guide.pdf]

Evaluating the Implementation and Effectiveness of Competency-Based Education in Sudanese Dental Curricula: A Comparative Analysis of Curriculum Models

Detailed Interview Guide for Semi-Structured Interviews

The following interview questions were developed based on an extensive literature review and expert input. They aim to explore participants' views on the implementation and effectiveness of competency-based education (CBE) in dental curricula, addressing four major domains:

Domain I: Curriculum Design and Implementation

1. **General Structure and Design**
    - Can you describe the overall structure of your institution's dental curriculum?
    - In what ways does the current curriculum reflect the principles of competency-based education?
2. **Strengths and Weaknesses**
    - What do you consider to be the key strengths of your curriculum in preparing students for clinical practice?
    - What aspects of the curriculum do you believe require improvement to better meet the demands of modern dental practice?
    - How well do you think the curriculum aligns with the intended learning outcomes and competencies?
3. **Curricular Fit for Purpose**
    - Do you feel that the curriculum adequately prepares students for real-world clinical challenges? Please explain.
    - Are there any specific courses or modules that you feel are particularly effective or ineffective? Why?

Domain II: Clinical Training and Practice

1. **Balance Between Theory and Practice**
    - How is hands-on clinical training integrated with theoretical instruction in your curriculum?
    - Do you feel that there is an appropriate balance between classroom-based learning and practical clinical experience? Provide examples.
2. **Effectiveness of Clinical Training**
    - In your experience, which components of clinical training have been most effective in enhancing student competencies?

- Are there elements within the clinical training portion that you believe could be improved? If so, how?

3. **Integration and Application**
    - How do the theoretical components of the curriculum translate into clinical practice according to your observations?
    - Can you describe any instances where the integration between what is taught in theory and the practical application was particularly successful or needs reconsideration?

Domain III: Interdisciplinary Collaboration and Communication

1. **Promotion of Interdisciplinary Learning**
    - How does your curriculum foster interdisciplinary collaboration among different dental specialties or with other healthcare disciplines?
    - Have you observed any benefits or challenges arising from cross-disciplinary learning initiatives?
2. **Development of Communication Skills**
    - In what ways are communication and interpersonal skills emphasized within the curriculum?
    - Can you provide examples of activities or assignments that specifically target the development of effective communication skills?
3. **Areas for Improvement**
    - Do you think there are missed opportunities for enhancing interdisciplinary learning or communication within the curriculum? Please elaborate.

Domain IV: Innovations in Dental Education

1. **Comparison of Educational Approaches**
    - How would you compare the current competency-based education approach with traditional teaching methods in dentistry?
    - What are the perceived benefits and drawbacks of the competency-based model as you see them?
2. **Impact of Innovations**
    - What innovative teaching methods or technologies have been introduced into your curriculum, and what impact have they had on student learning?
    - How have these innovations changed the way students prepare for clinical practice?
3. **Professional Readiness**

- In your opinion, how has the adoption of CBE influenced the overall professional readiness of dental graduates?
- Are there innovative practices or approaches that you believe should be further implemented to enhance educational outcomes and clinical preparedness?

General and Closing Questions

- What specific changes or enhancements would you propose to optimize the current curriculum for better competency development?
- Do you have any additional comments or suggestions regarding the integration of competency-based education in dental curricula?

This interview guide was pilot-tested with a small group of participants, and necessary revisions were made to improve clarity and relevance. The full interview topic guide is provided as supplementary material for transparency and reproducibility.
